# Synthesis and characterization of hydroxyapatite nanoparticles from calcium hydroxide fouled with gases evolved from smokestack of glass industry

**DOI:** 10.1038/s41598-024-60970-2

**Published:** 2024-05-14

**Authors:** Alaa Abdelmoaty, Sahar Mousa

**Affiliations:** Inorganic Chemistry Department, National Research Centre, 33 El Bohouth Street (Former Tahrir St.), Dokki, P.O. 12622, Cairo, Egypt

**Keywords:** Industrial waste, Calcium hydroxyapatite, Chemical treatment, Morphology, Porosity, Environmental sciences, Chemistry, Materials science, Nanoscience and technology

## Abstract

In glass industry, the evolved gases and fumes from burning the gas fuel absorbed in calcium hydroxide to minimize the pollution of environment. After a period of time, the calcium hydroxide fouled with sulphate and carbonate as action of the absorbed SO_3_ and CO_2_ gases. Based on our interest to treatment the solid waste materials, this study intended to convert the obtained waste of calcium hydroxide fouled with gases to valuable products. Firstly, this waste was treated with water, caustic soda and acids. The results confirmed the conversion of waste to pure calcium sulfate by treatment with 6 v/v% sulfuric acid. Secondly, the obtained calcium sulfate was reacted with ammonium dihydrogen phosphate solution for preparation of calcium hydroxyapatite (HAp) nanoparticles. The produced HAp sample was characterized by X-ray diffraction (XRD), Fourier transform infrared spectroscopy (FTIR), transmission electron microscopy (TEM) and scanning electron microscopy (SEM), thermal gravimetric analysis (TGA) and N_2_ adsorption measurements. The obtained findings confirmed that the HAp can be produced after calcination at 700 °C, nanorods-like of sizes ranged from 11 to 15 nm and with main surface functional groups of hydroxyapatite. TGA and DTA data indicated that HAp is thermally stable up to 700 °C. Also, the obtained HAp has Ca/P molar ratio of 1.60 and exhibited high total surface area of 146 m^2^/g with mesoporous structure which make this material can be used in medical and water purification applications.

## Introduction

Glass industries produce a lot of wastes ranging from the products of the used raw materials to the damaged glass products. Glass in general formed from a mixture of materials such as silicate, soda ash, and CaCO_3_ with other additives for coloring or improvement the properties of the end product by melting at high temperature followed by cooling to the solidification without crystallization. On the other hand, the used fuel during glass production generates a lot of waste. The fuel waste is more pollutant than that produced from the melting process^1^.

The main environmental challenges for the glass industry are polluting emissions and energy consumptions. Most of glass production used natural gas as a source of energy. The production of the glass passes through different stages of heating such as the decomposition of the start materials, melting and finishing. In general, the amount of pollution emission depends on the type of glass produced, the raw materials, the types of furnace and the fuel used. The recent literatures^2,3^ refer that the main evolved gas are CO, CO_2_ and NO_2_.

Unfortunately, various works^4–7^ were aimed to minimize the waste during the melting process, while a little attention was paid to manage the produced fumes and gases from the fuel combustion. In most cases, when the fuel burned produces carbon oxides, water vapor and some amounts of other dangerous gases. According to the regulation of the governments, the gases fuel should be removed or minimize the environmental pollution. The technology of glass manufacture refers that the waste fuel gases could be absorbed in some adsorbent materials such as Ca(OH)_2_^8^. After a period of work, the adsorbent becomes saturated and it should be replaced.

The exhausted Ca(OH)_2_ becomes a source of pollution, so that it is necessary to utilize the Ca(OH)_2_ which loaded with the fuel gases. In our previous works, several managements to minimize the pollution from the by-product of some chemical industries such as phosphogypsum produced from the phosphoric acid manufacture or cirtogypsum produced from citric acid or treatment the copper and zinc scrap were carried out^9,10^. As continuation of this strategy, the present work aims to convert the loaded Ca(OH)_2_ with gases to valuable materials. This study directed to produce high calcium content materials such as hydroxyapatite.

Hydoxyapatite (HAp), could be prepared from raw materials rich in Ca^2+^ and PO_4_^–3^ such as calcium carbonate, phosphate ores, phosphoric acid, ammonium dihydrogen phosphate and etc.^11^. Otherwise, HAp was prepared from some biogenic wastes such as egg shells, sea shells, animal bone and corals^12–15^.

HAp particles have several advantages such as Ca/P ratio equal 1.67 similar to that found in the human bones, thermal stability and high surface area. Owing to these properties it used in several applications such as biocompatibility, osteogenic ability and in removal of some pollutant elements from water. HAp used mainly in different biomedical applications such as implant coating, bone scaffold, bone filler and drug delivery. Moreover its structure can be grafted with special cations such as Ag and Zn to increase the bioactivity effects^16–19^.

## Experimental

### Materials

The solid waste, collected during one month of Ca(OH)_2_ which absorbed the evolved gases from combustion of the fuel, was supplied kindly from Guardian Glass Industry, 10th Ramadan city, Egypt. Caustic soda (99%, Merck), HCl (33% Adwic), H_2_SO_4_ (98% Adwic), ammonium dihydrogen phosphate (NH_4_)H_2_PO_4_, 99%, Merck), ammonia solution (26%Adwic) and distilled water were used without further purification.

### Methodology

#### Treatment of the waste

To determine the best method for waste treatment, the waste was treated by the following methods;

- Washed with water for several times
- Treatment with alkaline solution (10%)
- Treatment with HCl (50%) and H_2_SO_4_ (10%)
- Effect of sulfuric acid concentration ((2, 4, 6, 8 and 10%)

All the samples after treatment were separated, washed and exposed to chemical analysis by XRF technique (Table 1).

**Table 1:** XRF analysis of industrial waste, treated waste with distilled water (sample 1), alkaline solution (sample 2), HCl (sample 3) and H_2_SO_4_ (sample 4).

Main constituents (wt%)	SiO_2_	Al_2_O_3_	Fe_2_O_3_	MgO	CaO	Na_2_O	K_2_O	P_2_O_5_	SO_3_	F	Cl	LOI
Waste	0.64	0.11	0.06	0.25	23.13	21.63	0.30	0.14	38.59	0.45	3.98	10.51
Sample (1)	0.71	0.16	0.11	0.89	41.52	2.07	0.09	0.20	36.58	1.13	0.89	15.50
Sample (2)	1.45	0.35	0.21	3.55	47.79	0.22	0.02	0.40	5.17	2.79	0.13	37.66
Sample (3)	0.22	0.03	0.01	0.03	32.97	0.34	0.00	0.03	60.17	0.00	0.04	6.84
Sample (4)	0.15	0.04	0.06	0.04	33.19	0.34	0.00	0.03	59.60	0.00	0.01	0.48

*LOI*: loss on ignition at 1000 °C.

**a. Water treatment**

The waste (100 g) was washed with distilled water (500 ml) for three times by tap water, then filtered and dried at 100 °C (sample 1).

**b. Alkaline treatment**

The industrial waste was rinsed in a 10% caustic soda solution for one hour at room temperature, and the mixture was left for 24 h. Then the excess of caustic soda was removed by washing with tap water for several times until the pH of the filtrate become neutral. Then the treated sample was filtered and dried at 100 °C (sample 2).

**c. Acidic treatment**

The industrial waste was mixed separately in 50% of HCl (sample 3) and 10% of H_2_SO_4_ (sample 4) solutions for one hour at room temperature and the mixtures were left for 24 h. Then the excess acid solution was removed and the treated sample washed with tap water for several times until the pH of the filtrate become neutral. The treated samples were filtered, dried at 100 °C.

**d. Effect of sulfuric acid concentration**

**Figure 1:**
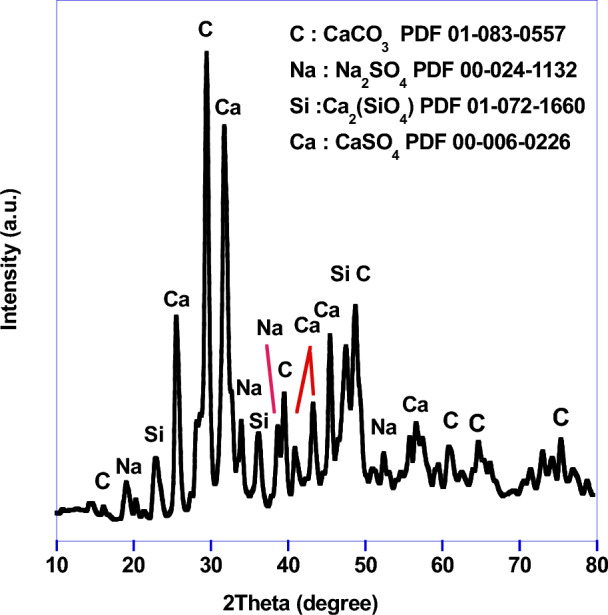
XRD pattern of waste sample.

The washed waste sample was treated with different concentrations of sulfuric acid (2, 4, 6, 8 and 10%) and calcined at 450 °C for 2 h to identify the best concentration. The produced phases were compared using XRD (Fig. 1).

#### Conversion the waste to hydroxyapatite

The conversion method was used elsewhere to convert the phosphogypsum to hydroxyapatite^17–19^. In this method, the treated waste with 6% H_2_SO_4_ (10 g) was suspended in amount of water (500 mL) under vigorous stirring at room temperature for 30 min. The required amount (5.0 g) of ammonium dihydrogen phosphate (NH_4_)H_2_PO_4_ solution was added dropwise with continue the stirring. The pH of the mixture was adjusted at 11 by using ammonia solution (18%) the chemical reaction was continued for 2 h. The solid part was separated by using centrifuge, dried at 100 °C and calcined at 700 °C for 2 h. The chemical reaction was preceded according to the following equation:

$${\text{5CaSO}}_{{4}} + {\text{3NH}}_{{4}} {\text{H}}_{{2}} {\text{PO}}_{{4}} + {\text{7NH}}_{{4}} {\text{OH}} \to {\text{Ca}}_{{5}} \left( {{\text{PO}}_{{4}} } \right)_{{3}} {\text{OH}} + {5}\left( {{\text{NH}}_{{4}} } \right)_{{2}} {\text{SO}}_{{4}} + {\text{6H}}_{{2}} {\text{O}}$$

### Characterization

The chemical compositions of industrial waste, all treated samples by water, alkaline and acidic solutions were analyzed by using Axios advanced Sequential WD_XRF Spectrometer, PANalytical2005 to quantify their percentages. The produced phases and crystalline nature of the prepared materials were studied using X-ray diffraction (XRD), Bruker (D8 advance) diffractometer (Germany) with copper (Ka) radiation which works at (40 kV and 40 mA) with 0.02°/0.4 s. The characteristic groups were measured by using JASCO–FT/IR-3000E infrared spectrometer from 4000 to 400 cm^−1^. The surface morphology of HAp was investigated by SEM (JEOL JXA-840A, Electron Probe Micro-Analyzer, Japan) at 20 kV. The shape and size of HAp nanoparticles were performed using high resolution-transmission electron microscope (HR-TEM, JEM-1230, Japan) operated at 200 kV. Thermal stability (TG and DTG) of HAp was performed using thermogravimetric analyzer (Shimadu TGA-50 H) under N_2_ flow over rate 30 ml/min at 10°/min). Brunauer–Emmett–Teller surface area (S_BET_, m^2^/g) and pore size distributions were measured using nitrogen adsorption analysis at − 196 °C (BEL-Sorp-max, Microtrac Bel Crop, Japan).

## Results and discussion

The waste sample was investigated using XRD (Fig. 1) where several compounds were occurred as CaCO_3_, CaSO_4_, Ca_2_(SiO_4_) and Na_2_SO_4_.To determine the content of each component and other minor compounds, XRF was carried out and the results were recorded in Table 1. The results show that the native waste contains CaO (23.13%) and SO_3_ (38.59%) with other oxides such as SiO_2_, Al_2_O_3_, Fe_2_O_3_ and etc. This means that Ca(OH)_2_ is partially converted to calcium sulfate and calcium carbonate by action of both sulfur oxide and carbon oxide gases which evolved from the fuel combustion. Thus, many trials were carried out to treat the industrial waste for eliminating all other impurities and to only one phase which used for synthesis of HAp.

For the effects of the treatment with water, alkaline and acids, it may be indicated that the washing with distilled water (sample 1) led to a drastic decrease from 21.63 to 0.09% in the percentage of sodium oxide. Moreover, an increase in the percentage of calcium from 23.13 to 41.52% and water content is obtained. This finding means that the sodium content dissolved in water so, the Ca content increases.

XRF results show that the treatment with 10% of caustic soda (sample 2) led to a considerable increase in the percentage of calcium from 41.52 to 47.79% while the SO_3_ decreases from 36.68 to 5.17%. The decrease in percentage of sulfate is due to the chemical reaction which occurred between sodium ions and sulphate ions.

The chemical treatment with HCl acid (sample 3) has negative effect where the calcium carbonate content converted to soluble calcium chlorides solution. In case of H_2_SO_4_ (sample 4), the carbonates converted into insoluble sulfates where theSO_3_% increased from 38.5 to 59.6% (Table 1). Hence, the acidic treatment with H_2_SO_4_ is the preferable usage to produce calcium sulfate as starting material for preparing HAp.

**Figure 2:**
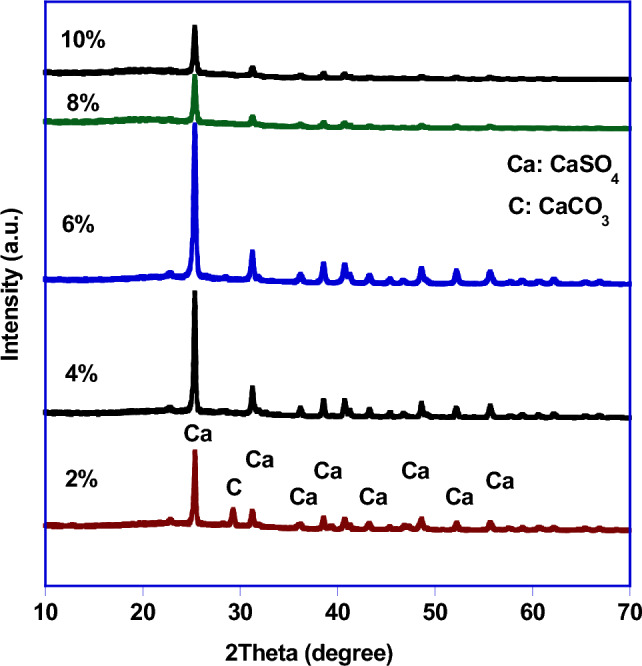
XRD patterns of treated industrial waste by different concentrations of H_2_SO_4_ (2, 4, 6, 8 and 10%).

Because sulfuric acid exhibited good results in the waste treatment process as mention above, a series of experiments were carried out to determine the best H_2_SO_4_ concentration. Figure 2 represents the XRD patterns of industrial waste after treatment with different concentrations of H_2_SO_4_ (2, 4, 6, 8 and 10 v/v%) and calcined at 450 °C for 2 h. It was obvious that the pattern of the sample which treated with 2% contains traces of CaCO_3_ (JCPDS (88-1809)) bedside the main phase of CaSO_4_ (JCPDS (72-0916)) while all the patterns of the other treated samples contains only one pure phase of CaSO_4_. Depending on the phase purity and good crystallinity, the best acid concentration for treatment was 6%.

**Figure 3:**
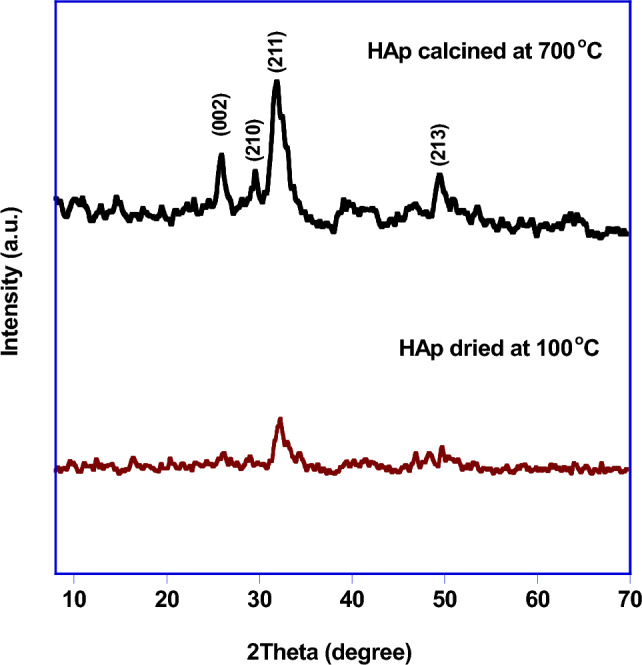
XRD patterns of HAp dried at 100 °C and calcined at 700 °C.

The obtained calcium sulfate from chemical reaction of solid waste with 6% sulfuric acid was used as calcium source to prepare hydroxyapatite (HAp). Figure 3 depicts the XRD patterns of the calcium hydroxyapatite which confirmed that HAp was obtained in weakly crystalline form. After calcination at 700 °C for 2 h, the calcium hydroxyapatite was formed as a monophasic material belonging to reference (JCPDS (76-0694)) with low crystallinity^20–22^.

**Figure 4:**
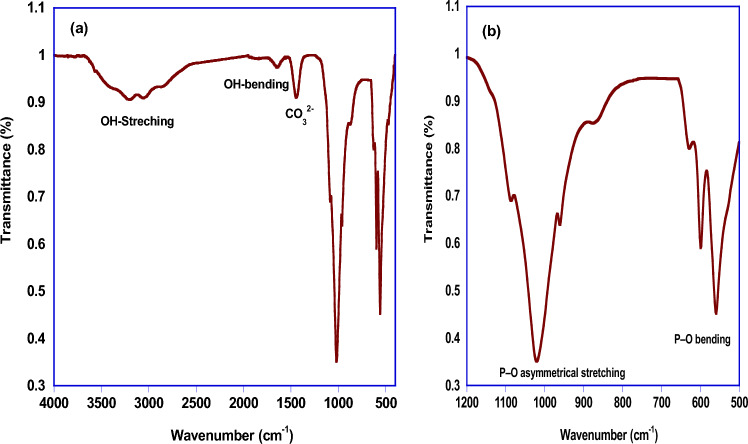
FTIR spectrum of HAp calcined at 700 °C.

Figure 4 illustrates the FTIR spectrum of HAp calcined at 700 °C where the characteristic functional groups of HAp are observed. The appearance of absorption bands at 3443–2851 cm^−1^ and at 1640 cm^−1^corresponds to O–H stretching and bending vibrational modes, respectively (Fig. 4a)^23–25^. The double bands at 606 and 561 cm^−1^ which attributed to the vibrational modes of phosphorous groups for HAp are appeared^26^. The band at 1022–1100 cm^−1^ which existed as a doublet or a shoulder is related to vibrational mode of P–O groups (Fig. 4b)^27–29^. While the weak band occurred at 1430 cm^−1^isascribed to the asymmetric stretching vibrations of CO_3_^2−^ (Fig. 4a). This finding indicates that partially carbonated hydroxyapatite can be formed during preparation process^30,31^.

**Figure 5:**
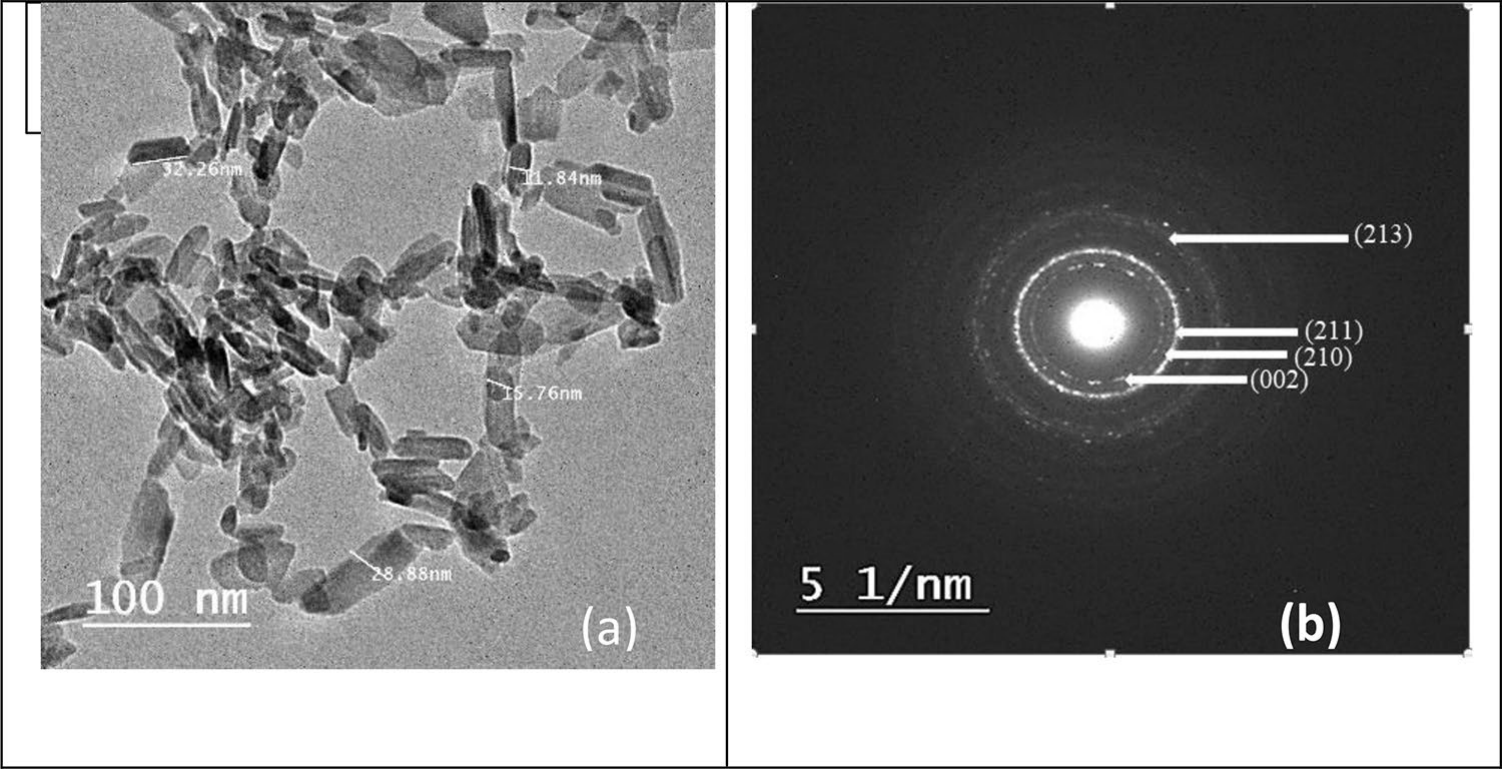
TEM image (**a**) and SAED pattern (**b**) of HAp calcined at 700 °C.

**Figure 6:**
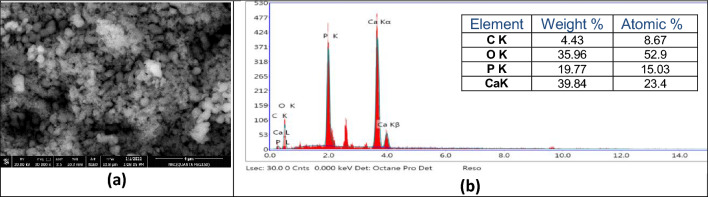
SEM micrograph (**a**) and EDX (**b**) of HAp calcined at 700 °C.

The morphological properties of HAp sample using TEM tool is presented in Fig. 5a. It is clear that hydroxyapatite formed in a nanostructure of rods-like shape (11–15 nm of thickness and 25–32 nm of length) which would candidate for many important applications in the science of bone tissue engineering. From the brightness and intensity of the corresponding selected area electron diffraction (SAED) pattern, the material is polycrystalline and the diffraction rings were assigned to the structure of pure hydroxyapatite (Fig. 5b). The SEM image of HAp sample is presented in Fig. 6a. It could be observed that the surface morphology of the sample was appeared as an ellipsoidal shape. The SEM micrograph emphasized that the HAp nanoparticles formed with high agglomeration as result of nanometric dimensions of the particles^32^. The elemental analysis of HAp was investigated by using (EDX) where the Peaks connected to Ca, P, and O confirmed that HAp formed with its necessary ions (Fig. 6b). The atomic ratio of Ca/P was 1.56 which is close to the standard stoichiometric ratio of the HAp (1.67).

**Figure 7:**
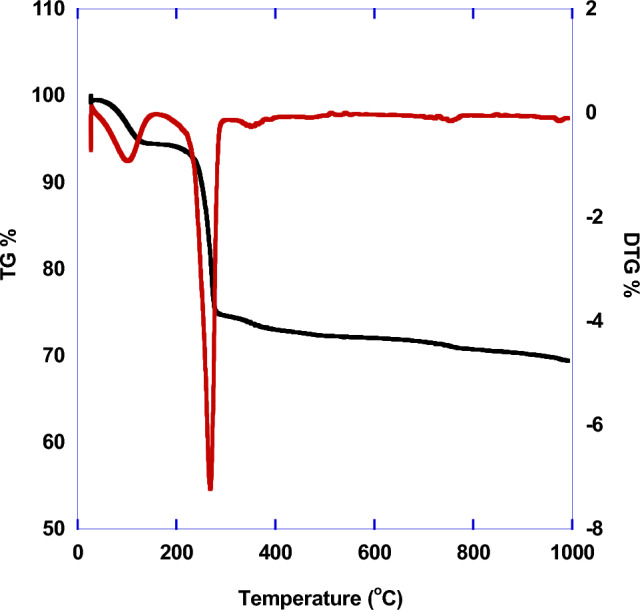
TG and DTG of prepared HAp.

The thermal gravimetric analysis for prepared HAp at 700 °C is illustrated in Fig. 7. The TGA and DTA profiles of HAp showed three regions. The first one is appeared at 155 °C with mass loss about 6% which corresponded to evaporation of moisture water and volatile matter. The second region at 290 °C with mass loss of 22% is due to the removal of residual ammonia. The last one at 790 °C with small mass loss of 4% is attributed to the removal of carbonates and water molecule as a result of partial conversion of HAp to tricalcium phosphate (TCP)^33^.

**Figure 8:**
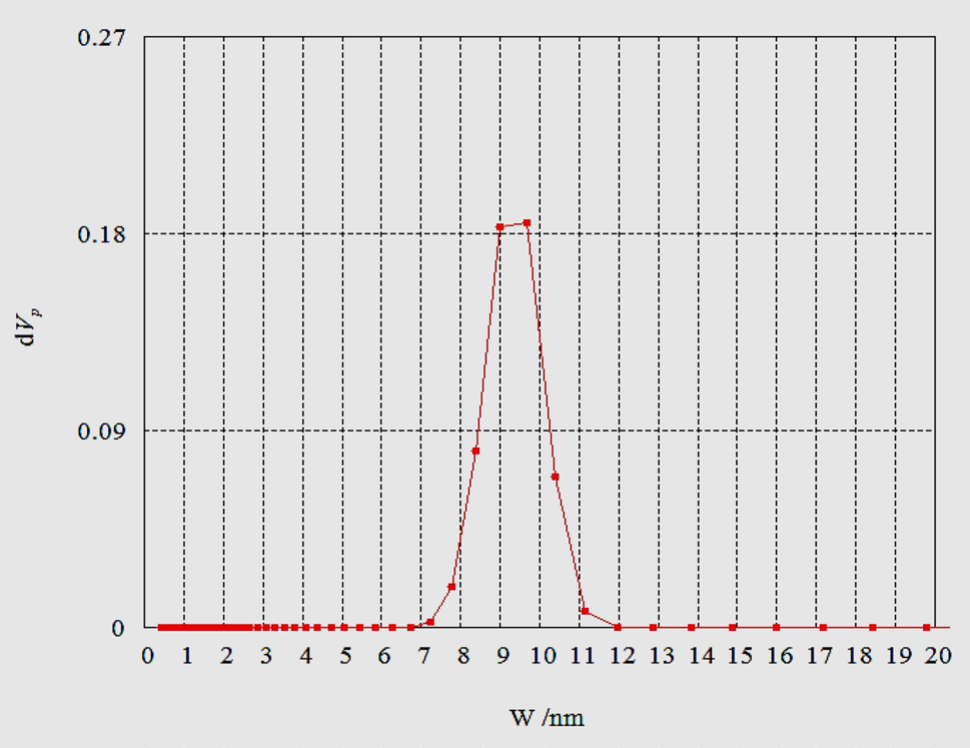
Pore size distribution analysis of HAp calcined at 700 °C by NLDFT.

In order to confirm the porous textures of HAp sample, N_2_ gas adsorption was performed. The measured BET surface area was found to be 146 m^2^/g with pore diameter (16.3 nm) and total pore volume (0.593 nm). The pore size distribution analysis according to NLDFT theory is illustrated in Fig. 8. It is noted that the maximum pore size of the HAp sample is mainly centered at 9.6 nm, confirming that the prepared sample has mesoporous structure^34,35^.

## Conclusions

From the analysis of the experiments and characterization of the prepared products, it may conclude that:

1. The Ca(OH)_2_ waste loaded with carbon oxide and sulfur oxide which results from combustion of fuel gas could be converted to pure calcium sulphate by action of 6 v/v%H_2_SO_4_.
2. The obtained calcium sulphate could be converted completely to HAp nanoparticles (11–15 nm of thickness and 25–32 nm of length) by reaction with ammonium dihydrogen phosphate at alkaline medium and calcination at 700 °C for 2 h as confirmed by XRD, FTIR, SEM and TEM analyses.
3. The composition of prepared HAp was investigated by EDX technique where the essential elements of HAp (O, Ca and P) were occured and the Ca/P ratio was 1.56, indicating that the prepared HAp is closely to natural HAp that exists in the human bone structure.
4. The produced HAp exhibited high surface area (146 m^2^/g) and a mesoporous structure which can candidate for medical and water purification applications.

Therefore, this work can be considered as a case study for achieving environmental and economic aspects through converting industrial wastes of glass industry to valuable materials.

## Data Availability

The data that support the findings of this study are available on request from the corresponding author, Alaa Abdelmoaty. The data are not publicly available due to their containing information that could compromise the privacy of research participants.

## References

[1] Gowtham R, ManikandaPrabhu S, Gowtham M, Ramasubramani R (2021). A review on utilization of waste glass in construction field. Mater. Sci. Eng..

[2] Michael Z, Peter S, Leander K, Detlef S (2021). A review of decarbonization options for the glass industry. Energy Convers. Manag. X.

[3] Cristian B, Jorge P, Manuel B, Begona P (2023). Techno-economic assessment of glassmaking decarbonization through integration of calcium looping carbon capture and power-to-gas technologies. Sustain. Prod. Consum..

[4] Ogundairo TO, Adegoke DD, Akinwumi II, Olofinnade OM (2019). Sustainable use of recycled waste glass as an alternative material for building construction: A review. IOP Conf. Ser. Mater. Sci. Eng..

[5] Malek M, Lasica W, Jackowski M, Kadela M (2020). Effect of waste glass addition as a replacement for fine aggregate on properties of mortar. Materials.

[6] Mao L, Zhang W (2018). Addition of waste glass for improving the immobilization of heavy metals during the use of electroplating sludge in the production of clay bricks. Constr. Build. Mater..

[7] Spiesz P, Rouvas S, Brouwers H (2016). Utilization of waste glass in translucent and photocatalytic concrete. Constr. Build. Mater..

[8] Caputo A, Pelagagge P (1999). Cost-effectiveness analysis of waste gas treatment plants for the glass industry. J. Air Waste Manag. Assoc..

[9] Baruaa E, Deoghare A, Deb P, Lala S, Chatterjee S (2019). Effect of Pre-treatment and Calcination Process on Micro-Structural and Physico-Chemical Properties of Hydroxyapatite derived from Chicken Bone Bio-waste. Mater. Today. Proc..

[10] El Rafie G, El Ghytany S, Ramadan R, Gaber M (2019). Treatment and purification of phosphogypsum. Egypt. J. Chem..

[11] Mohamed K, Mousa S, Abd El-Hady B, Tolba E, El Bassyouni G (2022). Fabrication of hydroxyapatite–aluminum silicate/chitosan-gelatin biocomposites with in-vitro application by preosteoblast cells (MC3T3-E1). Egypt. J. Chem..

[12] Baba A, Oduwole I, Salami F, Adekola F, Adeboye S (2013). Synthesis of hydroxyapatite from waste egg-shell by precipitation method. J. Life sci..

[13] Cahyanto A, Kosasih A, Aripin D, Hasratiningsih Z (2017). Fabrication of hydroxyapatite from fish bones waste using reflux method. Mater. Sci. Eng..

[14] Mohana N, Palangadanb R, Fernandeza F, Varmaa H (2018). Preparation of hydroxyapatite porous scaffold from a ‘coral-like’ synthetic inorganic precursor for use as a bone substitute and a drug delivery vehicle. Mater. Sci. Eng. C.

[15] Sobczak A, Kowalski Z, Wzorek Z (2009). Preparation of hydroxyapatite from animal bones. Acta Bioeng. Biomech..

[16] Diana G, Vasile-Adrian S, Andrei P, Ecaterina A (2022). Current development in biomaterials-hydroxyapatite and bioglass for applications in biomedical field: A review. J. Funct. Biomater..

[17] Mousa S, Hanna A (2013). Synthesis of nano-crystalline hydroxyapatite and ammonium sulfate from phosphogypsum waste. Mater. Res. Bull..

[18] Mohamed K, Mousa S, El- Bassyouni G (2014). Fabrication of nano structural biphasic materials from phosphogypsum waste and their in vitro applications. Mater. Res. Bull..

[19] Mousa S, Ammar N, Ibrahim H (2016). Removal of lead ions using hydroxyapatite nano-material prepared from phosphogypsum waste. J. Saudi Chem. Soc..

[20] Moaness M, Mousa SM, Abo-Elfadl MT, El-Bassyouni GT (2024). Doxorubicin loaded cerium substituted hydroxyapatite nanoparticles: A promising new therapeutic approach for bone regeneration, doxorubicin delivery, and cancer treatment. Int. J. Pharm..

[21] Ghedjemis A, Benouadah A, Fenineche N, Ayeche R, Hatim Z, Drouiche N, Lounici H (2019). Preparation of hydroxyapatite from dromedary bone by heat treatment. Int. J. Environ. Res..

[22] Ghedjemis A, Ayeche R, Benouadah A, Fenineche N (2021). A new application of hydroxyapatite extracted from dromedarybone: Adsorptive removal of Congo red from aqueous solution. Int. J. Appl. Ceram. Technol..

[23] Mabrouk M, Mousa S, Abd ElGhany W, Abo elfadl M, El Bassyouni G (2021). Bioactivity and cell viability of Ag^+^ and Zr^4+^ co-doped biphasic calcium phosphate. Appl. Phys. A..

[24] Siek D, Ślósarczyk A, Przekora A, Belcarz A, Zima A, Ginalska G (2017). Evaluation of antibacterial activity and cytocompatibility of α-TCP based bone cements with silver-doped hydroxyapatite and CaCO_3_. Ceram. Int..

[25] Vecstaudza J, Gasik M, Locs J (2019). Amorphous calcium phosphate materials: Formation, structure and thermal behaviour. J. Eur. Ceram. Soc..

[26] Elsayed E, El-Ashmawy A, El-Bassyouni G, Mousa S, Manawaty M, Emara L (2023). Formulation and evaluation of alginate-gelatin hydrogel scaffolds loaded with zinc-doped hydroxyapatite and 5-fluorouracil. Int. J. Biol. Macromol..

[27] Fu C, Zhang X, Savino K, Gabrys P, Gao Y, Chaimayo W (2016). Antimicrobial silver-hydroxyapatite composite coatings through two-stage electrochemical synthesis. Surf. Coat. Technol..

[28] Daryan S, Javadpour J, Khavandi K, Erfan M (2018). Morphological evolution on the surface of hydrothermally synthesized hydroxyapatite microspheres in the presence of EDTMP. Ceram. Int..

[29] Laonapakul T (2015). Synthesis of hydroxyapatite from biogenic wastes. Eng. Appl. Sci. Res..

[30] Ghedjemis A, Ayeche R, Kebaili M, Benouadah A, Gil LF (2022). Application of natural hydroxyapatite in the treatment ofpolluted water: Utilization of dromedary bone as bioadsorbent. Int. J. Appl. Ceram. Technol..

[31] Ghedjemis A, Ayeche R, Benouadah A (2022). A comparative study on proprieties of hydroxyapatite prepared from bovine and dromedary bone. J. Aust. Ceram. Soc..

[32] Stanic V, Janackovic D, Dimitrijevic S, Tanaskovic B, Mitric M, Pavlovic S (2011). Synthesis of antimicrobial monophase silver-doped hydroxyapatite nanopowder for bone tissue engineering. Appl. Surf. Sci..

[33] Supová M (2015). Substituted hydroxyapatites for biomedical applications: A review. Ceram. Int..

[34] Gunduz O (2014). A simple method of producing hydroxyapatite and tri calcium phosphate from coral (*Pocillopora verrucosa*). J. Aust. Ceram. Soc..

[35] Wan Y, Zhao D (2007). On the controllable soft templating approach to mesoporous silicates. Chem. Rev..

